# Target volume definition in high-risk prostate cancer patients using sentinel node SPECT/CT and ^18^ F-choline PET/CT

**DOI:** 10.1186/1748-717X-7-134

**Published:** 2012-08-08

**Authors:** Hansjörg Vees, Charles Steiner, Giovanna Dipasquale, Amine Chouiter, Thomas Zilli, Michel Velazquez, Sophie Namy, Osman Ratib, Franz Buchegger, Raymond Miralbell

**Affiliations:** 1 Division of Radiation Oncology, University Hospital, Geneva, Switzerland; 2 Division of Nuclear Medicine, University Hospital, Geneva, Switzerland; 3 Division of Radiology, University Hospital, Geneva, Switzerland

**Keywords:** Prostate cancer, Radiotherapy, SPECT, Sentinel node, ^18^ F-choline PET/CT

## Abstract

**Background:**

To assess the influence of sentinel lymph nodes (SNs) SPECT/CT and ^18^ F-choline (^18^ F-FCH) PET/CT in radiotherapy (RT) treatment planning for prostate cancer patients with a high-risk for lymph node (LN) involvement.

**Methods:**

Twenty high-risk prostate cancer patients underwent a pelvic SPECT acquisition following a transrectal ultrasound guided injection of ^99m^Tc-Nanocoll into the prostate. In all patients but one an ^18^ F-FCH PET/CT for RT treatment planning was performed. SPECT studies were coregistered with the respective abdominal CTs. Pelvic SNs localized on SPECT/CT and LN metastases detected by ^18^ F-FCH PET/CT were compared to standard pelvic clinical target volumes (CTV).

**Results:**

A total of 104 pelvic SNs were identified on SPECT/CT (mean 5.2 SNs/patient; range 1–10). Twenty-seven SNs were located outside the standard pelvic CTV, 17 in the proximal common iliac and retroperitoneal regions above S1, 9 in the pararectal fat and 1 in the inguinal region. SPECT/CT succeeded to optimize the definition of the CTV and treatment plans in 6/20 patients due to the presence of pararectal SNs located outside the standard treatment volume. ^18^ F-FCH PET/CT identified abnormal tracer uptake in the iliac LN region in 2/19 patients. These abnormal LNs were negative on SPECT/CT suggesting a potential blockade of lymphatic drainage by metastatic LNs with a high tumour burden.

**Conclusions:**

Multimodality imaging which combines SPECT/CT prostate lymphoscintigraphy and ^18^ F-FCH PET/CT identified SNs outside standard pelvic CTVs or highly suspicious pelvic LNs in 40% of high-risk prostate cancer patients, highlighting the potential impact of this approach in RT treatment planning.

## Background

Prostate cancer patients with a high-risk of nodal involvement are most frequently treated with an association of androgen deprivation and radiotherapy (RT) to the prostate, seminal vesicles, and pelvic lymph nodes (LNs) [1,2]. The consensus guidelines of the Radiation Therapy Oncology Group (RTOG) recommend the irradiation of the distal common iliac, presacral (S1-S3), external iliac, internal iliac, and obturator LN regions [3]. However, lymph from the prostate may also drain to nodes in the pararectal, internal pudendal, and anterior abdominal regions [4,5].

Lymphoscintigraphy of the prostate with ^99m^Tc-Nanocoll enables the detection of sentinel lymph nodes (SNs) with a very high sensitivity of 93% to 98% [5,6]. Different studies have shown that registered images of SPECT and CT can detect more SN than a lymphoscintigraphy alone [7,8]. Although, there is lack of consensus regarding the value of PET imaging in the primary diagnostic of prostate cancer, PET/CT using ^18^ F- or ^11^C-labeled acetate or choline has shown a high potential in visualizing local and regional recurrence in prostate cancer with a high sensitivity and specificity [9].

The aim of this study was to evaluate the potential of SN SPECT/CT in RT treatment planning in prostate cancer patients with a high-risk for LN involvement. As no pathological verification of the SNs was planned in this study, a ^18^ F-choline (^18^ F-FCH) PET/CTs was performed in order to get additional information on the potential metastatic infiltration of the SPECT positive SNs.

## Methods

This prospective study was approved by the Ethical Committee at the Geneva University Hospital. A signed informed consent was obtained from all patients participating in the study. Given that ^18^ F-FCH tracer is not registered in Switzerland, it was administered under the ruling of “special use”, which requires approval on a patient by patient basis by the Swiss federal authorities (Swissmedic and the Federal Office of Public Health, Section of Radioprotection).

Twenty prostate cancer patients were included in the study from May 2007 to April 2009. Eligible patients were selected based on a biopsy-confirmed adenocarcinoma of the prostate with a high risk for LN metastases. The estimated risk for pelvic LN involvement was ≥20% according to the predictive model developed by Roach et al. [10]. A negative metastatic work-up was obtained for every patient before entering the study with an abdominal CT, and a bone scan. After entering the study, all patients underwent a prostate SPECT/CT lymphoscintigraphy and all but one patient had an additional ^18^ F-FCH PET/CT. The decision to incorporate ^18^ F-FCH PET/CT in the study was taken according to an amendment to the study established after the inclusion of the first patient.

The median age was 67 years (range, 51–74) and the median PSA at diagnosis was 30.5 ng/ml (range,6.4–66.5). The clinical characteristics and referral patterns of the patient population are summarized in Table 1.

**Table 1:** Disease characteristics of the patient population in the study (n = 20)

**Characteristics**	**Number (%)**
Initial stage	
cT1c	2 (10)
cT2	2 (10)
cT3a	5 (25)
cT3b	11 (55)
cN0	20 (100)
PSA at diagnosis (ng/ml)	
< 10	6 (30)
10–20	6 (30)
> 20	8 (40)
Initial Gleason score	
6	1 (5)
7	9 (45)
8–9	10 (50)
Roach score	
20–35%	13 (65)
35–50%	6 (30)
>50%	1 (5)
Perineural infiltration	
No	6 (30)
Yes	14 (70)

Abbreviation: PSA = prostate-specific antigen.

### Lymphoscintigraphy technique and SPECT/CT fusion imaging and interpretation

Four aliquots, of 50 MBq in 1 mL ^99m^Tc-nanocolloid (Nanocoll1, GE Healthcare, Amersham Health, Piscataway, NJ) were injected into both lobes of the prostate using a Chiba needle (0.70 x 220 mm) under transrectal ultrasound guidance (Toshiba APLIO SSA-770A) applying 1 fraction in the apical and 1 in the basal part of each lobe. All injections were performed by an experienced radiologist (AC) under antibiotic coverage. Spill of the radiocolloid into the rectum could be avoided. All patients were asked to empty their bladder after the injection. Immediately before the imaging, the patients’ skin was marked with 3 fiducial body markers (Multi-Modality NucMed/PET Markers MM3003, IZI Medical Products, Baltimore, MD) containing 0.1–0.2 MBq of ^99m^TcO_4_ (pertechnetate) over the pelvis. After early planar images, SPECT of the pelvis and abdomen (γ camera, IRIX, Philips, Milpitas, CA) was performed 90 minutes after tracer injection. All distinctly visible LNs on SPECT were defined as SNs.

The SPECT imaging was performed on a dedicated RT table in the same position as the treatment planning PET/CT or CT for the one patient who didn’t had the PET/CT. The acquisition protocol for SPECT was standardised (3 Head Gamma Camera Irix (Marconi), LEGAP collimator, 60 frames of 60 sec. each in steps of 6°, 128 x 128 matrix) as was the image post processing (count truncation of injection site maxima, iterative reconstruction (OSEM), 4–20 iterations, Butterworth post filtering, reconstructed slice thickness 4.7 mm). Reconstructed transverse data sets were overlain with the corresponding CT. Image fusion was performed by the use of body markers using commercially available software (Syngo 3D Fusion TM, Siemens, Erlangen, Germany). The fusion accuracy (5 mm) and the 99mTc-activity calibration were experimentally validated by preceding phantom studies. SPECT calibration was performed on an object of similar shape and size as the simulated object and surrounding tissues (EMA IEC/2001 image quality body phantom) with spherical inserts suspended by plastic rods. The spheres were filled with ^99m^Tc-nanocolloid activity with ascending concentration ranging from 0.7–2.8 MBq. In all cases, image fusion of SPECT and CT was performed by the same nuclear medicine specialist (CS). Images were read by two examiners in consensus reading to determine the number of SNs, pelvic side, and anatomic region according to the nodal atlas of Martinez-Monge et al. [11]. Each sentinel node was defined in transverse, sagittal, and coronal views.

### ^18^ F-fluorocholine PET/CT scanning

GMP ^18^ F-fluorocholine (fluorocholine-fluoromethyl-dimethyl-2-hydroxyethyl-ammonium) was obtained from the Centre of Radiopharmacy, University Hospital of Zürich, Switzerland.

All but one patient had a PET/CT on a LSO-based PET/CT scanner (Biograph 16, Siemens Medical Solution, Erlangen, Germany) operating in 3D mode. All patients were fasted for at least four hours before the ^18^ F-FCH PET/CT. The ^18^ F-FCH PET/CT was performed in RT treatment position on a dedicated RT table equipped with a set of three triangulation lasers (central and laterals), and intrinsic supports units similar to those used for standard CT-based virtual simulation. After the CT scan, patients were injected 300 MBq ^18^ F-fluorocholine and immediately underwent a continuous list-mode PET acquisition of the pelvis during 10 minutes. Following the list mode acquisition a standard whole-body PET study was performed from the mid thigh to the skull over 7 to 8 bed positions of 3 to 4 minutes each, depending on patient size and weight. Two additional late PET acquisitions of 5 minutes of prostate bed and adjacent pelvic region were acquired immediately after whole-body PET (~40 minutes after tracer injection). A detailed description of the PET scanning technique has been previously published by our group [12].

### RT planning

Clinical target volumes (CTV) were drawn on series of CT slices by an experienced radiation oncologist and were defined according to the RTOG guidelines on pelvic lymph node volumes [3]. The CTV included the prostate, the seminal vesicles, the distal common iliac, presacral, external iliac, internal iliac, and obturator LNs. The obturator and internal iliac LNs were contoured from the obturator foramen up to the distal common iliac vessels at the level of the upper aspect of the L5/S1 interspace. The external iliac LNs were drawn from the level where the external iliac vessels cross the inguinal ligament to the same upper level of the internal iliac nodes. The presacral LNs were drawn between S1 and S3. Organs at risk were delineated as well using the CT images, and included the rectum, the bladder, the bowel, and both femoral heads. SPECT images were highlighted and coregistered with the CT.

### Statistical analysis

Statistical analyses and curve fitting were performed using SPSS® (version 15.0, SPSS Inc., Chicago, IL, USA). The level of statistical significance was 0.05.

## Results

Both lymphoscintigraphy and ^18^ F-FCH PET/CT studies were well tolerated with no toxicity. A total of 104 SNs were detected after the injection of ^99m^Tc-nanocolloid, thus a mean of 5.2 LN/patient (range, 1–10). SNs drained most frequently to external iliac and common iliac regions (Table 2). In 14 patients, however, 27 SNs were located outside the pelvic CTV region: nine pararectal, 1 right inguinal and 17 in the retroperitoneal region above S1. Patients with retroperitoneal SNs presented in addition a median of 7 (range, 3–10) SNs in the pelvis compared to a median of only 3 (range, 1–5) pelvic SNs in those patients with no SNs in the retroperitoneal region (Table 2). The remaining 77 SNs were all included in the CTV (see Table 3). All SNs were non-suspicious according to metabolic and morphologic criteria: normal size and shape, and negative ^18^ F-FCH uptake.

**Table 2:** Number and localization of sentinel lymph nodes according to the different pelvic lymph node regions

**Pat. No**	**internal pudendal**	**SV**	**inguinal**	**para-rectal**	**Pre-sacral**	**obturator**	**internal iliac**	**external iliac**	**common iliac**	**Left para-aortic**	**Right para-aortic**	**Total**
1				1		1		1				**3**
2				1				2				**3**
3					1		1		2	2	1	**7**
4		1					1	2	1	1	1	**7**
5						1		2	2	1	2	**8**
6					2	1	1		1	1		**6**
7				1			1		1		1	**4**
8								1	1		1	**3**
9				1	2			2				**5**
10								3				**3**
11					1	2		2	1		2	**8**
12			1				2	2	3	1		**9**
13		1		1		1			3	1		**7**
14						2		2				**4**
15				2	1	1		3	2		1	**10**
16					1			1	1	1		**4**
17				1		1		1				**3**
18						1						**1**
19					1		2	2				**5**
20				1				2	1			**4**
**Total**	**0**	**2**	**1**	**9**	**9**	**11**	**8**	**28**	**19**	**8**	**9**	**104**

Abbreviation: SV: seminal vesicle lymph plexus.

**Table 3:** Localization of sentinel lymph nodes in relation to the pelvic CTV

**SN**	**Inside CTV**	**Outside CTV**
SV	2	0
Inguinal	0	1
Pararectal	0	9
Presacral	9	0
Obturator	11	0
Internal iliac	8	0
External iliac	28	0
Distal common iliac	19	0
Left paraaortic	0	8
Right paraaortic	0	9
Total	77	27

Abbreviation: VS: seminal vesicle lymph plexus.

**Figure 1:**
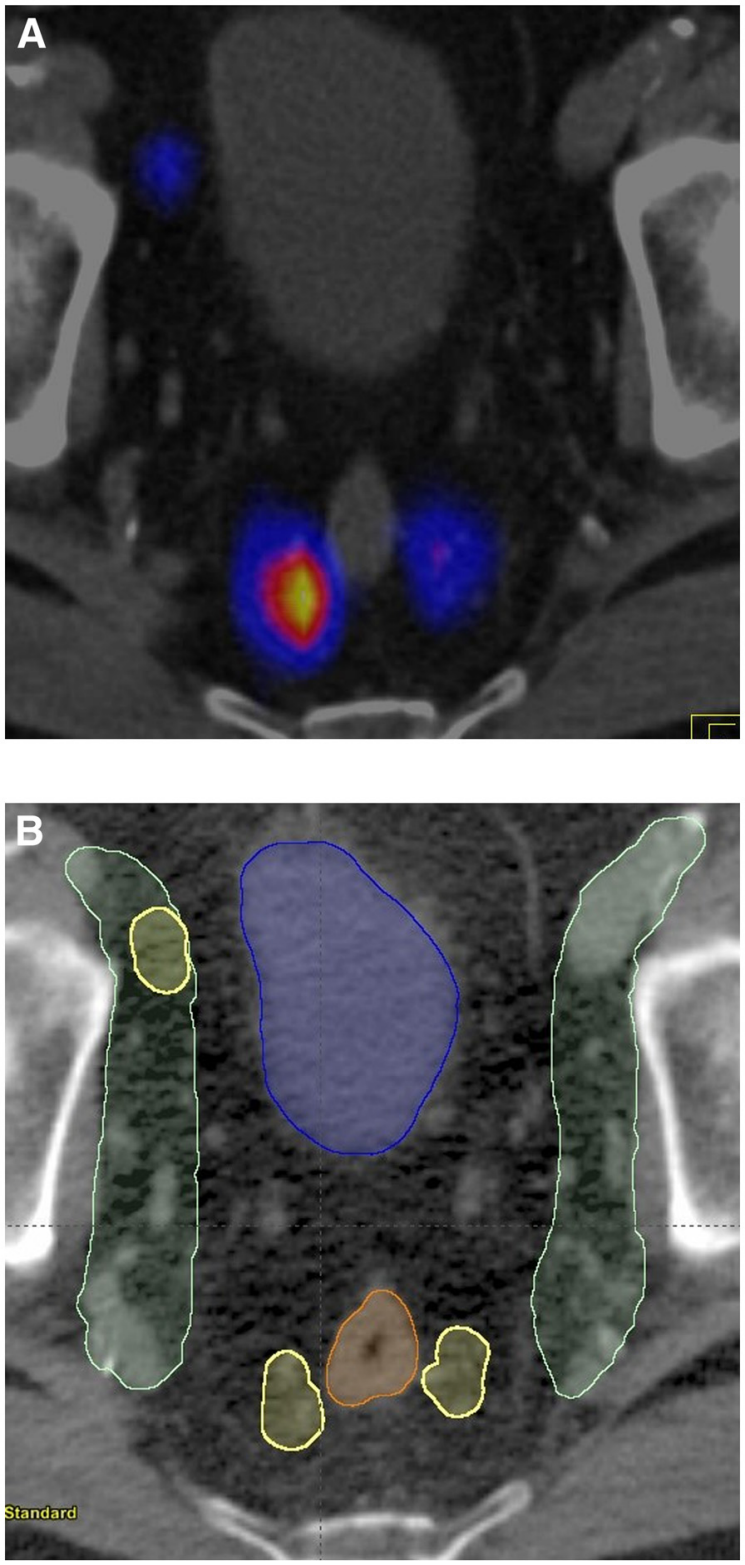
**(A) Fusion image of SPECT and planning CT (patient no. 6) showing a transverse section of two pararectal and a right external iliac SN.** (**B**) Axial view of the standard nodal clinical target volume (CTV) (green) and the pararectal SNs (yellow).

The location of the SNs was evaluated according to a 4-field pelvic standard treatment plan. The paraaortic and the right inguinal SNs were disregarded for treatment planning. Two of the pararectal SNs received at least 95% of the prescribed radiation dose (patient no. 1 and 9). The remaining SNs found in the pararectal region and outside the original CTV were included in an enlarged new CTV. They accounted for 7 SNs in 6 patients (30%). A fusion image of SPECT and planning CT of a pararectal SN is shown in Figure 1.

**Figure 2:**
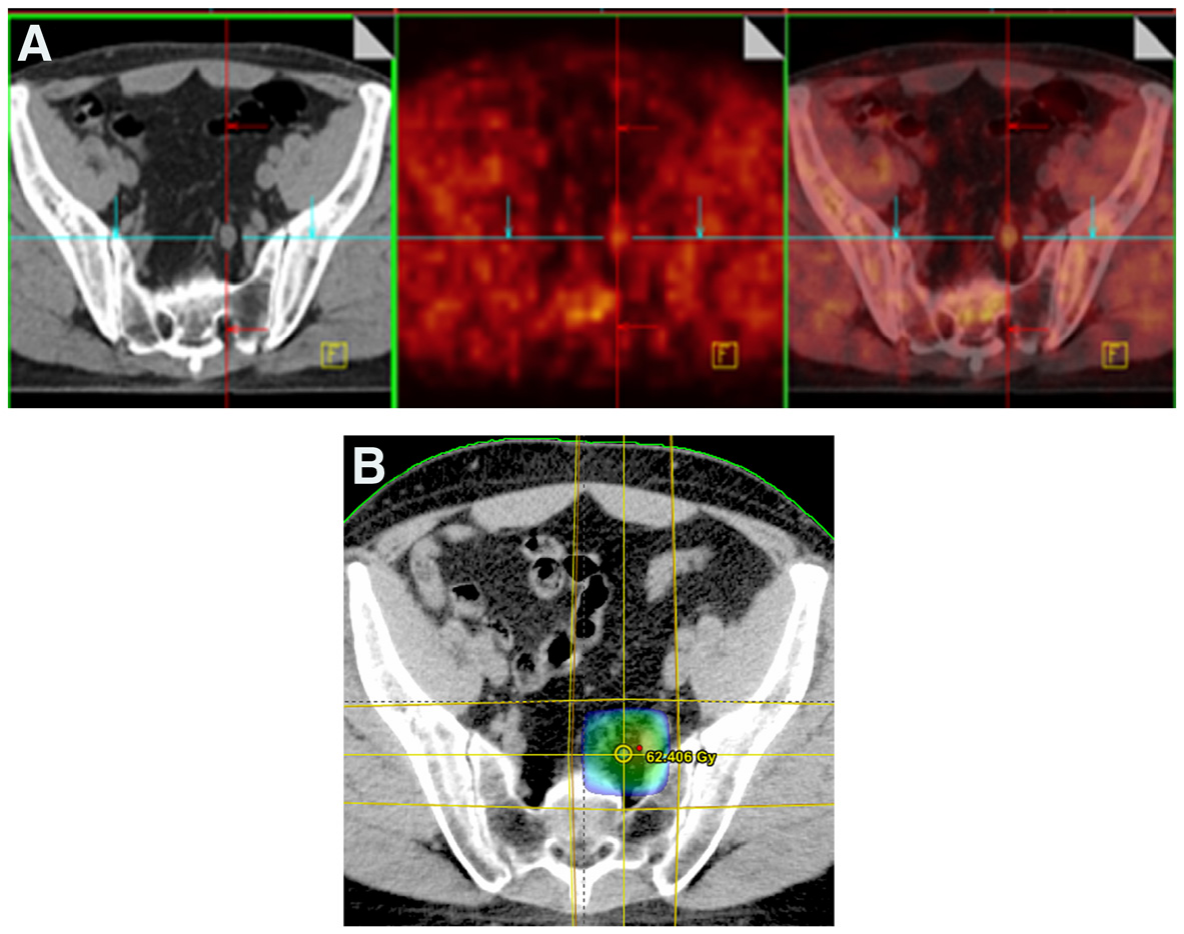
**(A)**^**18**^ **F-FCH PET/CT (patient no. 3) showing a transverse section with a suspicious lymph node metastasis in the left internal iliac region.** (**B**) The corresponding color wash dose distributions for the planning target volume (PTV).

In all patients but two undergoing ^18^ F-FCH PET/CT studies no suspected regional LN metastases were detected. In the two patients with an abnormal tracer uptake in the LN region, the first one (patient no. 3) had a suspect LN metastasis in the left internal iliac area with a SUV_max_ of 2.6 and the second one (patient no. 9) had a suspect LN metastasis in the right external iliac area with a SUV_max_ of 4.5. SPECT/CT studies did not show any ^99m^Tc-uptake in both suspected metastatic LNs based on ^18^ F-FCH PET/CT studies probably because of a lymphatic drainage blockade secondary to a massive tumour invasion of the concerned LNs. The suspected LN metastases were both located inside the standard CTV and were treated with an additional boost dose (Figure 2). All ^18^ F-FCH PET/CT studies were negative for distant disease.

## Discussion

Most SNs in our study were located inside a standard CTV for pelvic LNs. However, 27 SNs were detected outside the CTV and only 2 were expected to receive at least 95% of the prescribed radiation dose with a 4-field pelvic 3D-CRT box technique. Indeed, 14 patients (70%) presented with SNs outside the pelvic CTV region. Unlike Ganswindt et al. and Krengli et al. we observed a relatively higher number of SNs in the retroperitoneal region [6,13]. Indeed, paraaortic SNs were observed in 55% of our patients. One patient presented a SN in the right inguinal region. The SNs in the retroperitoneal region and in the right inguinal bed were excluded from the treatment volume as described above. Wengenmair et al. showed that the accumulation of ^99m^Tc in LNs is highly variable and is generally completed 90 minutes after tracer injection [14]. We defined the SNs in the SPECT according to their results and found a higher number of SNs in those patients with the presence of retroperitoneal SNs. This period is maybe too long in some cases and several of the positive LNs in the SPECT are no longer part of the first step lymph drainage of the prostate but rather a second step drainage. The results of a retrospective study by Vermeeren et al. were in concordance with this hypothesis [15]. They showed that the incidence of LN metastases in paraaortic SNs is low. Treating the paraaortic region prophylactically based on SN positivity may not be beneficial but may rather increase dangerously the risk of radiation induced intestinal toxicity. However, a drawback of this study is the lack of pathological verification of the SNs.

The appearance of inguinal LN metastasis from prostate cancer is very rare and may occur mostly in patients with advanced metastatic disease [16]. SNs in the inguinal region have only been described in a series of 3 patients with SNs in the ventral abdominal wall and in only one of these patients the presence of tumour was confirmed on pathological examination [4]. In our study only one suspicious inguinal SN was observed. This patient had a surgical intervention for inguinal hernia with potential subsequent disruption and rerouting of lymphatic pathways resulting in an aberrant lymphatic drainage to the inguinal LN region and was not included in the treatment volume. We did not observe SNs in the internal pudendal region. The published incidence of SN in the internal pudendal region is very low, ranging between 1–2% [6].

Serum PSA, clinical stage, and Gleason score are widely recognized predictors of aggressiveness of prostate cancer. In patients with a high-risk profile, the inclusion of the pelvic LNs in the RT treatment volume may improve the rate of disease free survival compared to those treated to the prostate alone [10]. The prostate SN as the first lymphatic echelon receiving lymph drainage is the most likely site for early metastases, especially in high-risk patients. Although, SPECT/CT lymphoscintigraphy has shown a high sensitivity in detecting SNs, there is at the present time no recommendation even for high-risk prostate cancer patients to irradiate the whole lymph drainage of the prostate. Indeed, the inclusion of all possible lymph drainage would increase the treatment volume excessively and would represent a too high-risk of radiation induced intestinal and urinary toxicity. The RTOG trial 75–06 was unable to detect significant differences in prostate cancer patients with detectable disease confined to the pelvis and treated with or without elective paraaortic irradiation [17].

Treatment optimization in RT offers the possibility of highly conformal radiation dose distribution by using techniques such as intensity-modulated RT (IMRT), which is superior to straight three-dimensional conformal RT in limiting the volume of bowel, bladder and rectum within the high- to intermediate-dose region opening the potential for dose escalation [18]. The delivery of a prophylactic irradiation dose to the distant paraaortic nodes can be safely performed using IMRT technique. One of the known drawbacks of IMRT technique is that it may increase the risk of geographical miss of LNs, which are outside the recommended CTV. The evaluation of SN SPECT imaging in prostate cancer patients has shown that SNs may be excluded from the CTV in 25–76% of cases. This risk may be even higher when treating with IMRT [6,13].

The inclusion of pelvic SNs in the CTV for prostate cancer patients with a high risk of LN metastases offers the opportunity to better adapt to the individual variability of the lymph drainage and may minimize the risk of missing the irradiation of metastatic LNs. Transrectal injection of ^99m^Tc-Nanocoll in the prostate guided by ultrasound is feasible and without major complications and implies the possible indication for this approach in high-risk prostate cancer patients. One may consider a drawback of this study that a post-acquisition co-registration between SPECT and CT images was performed. However both imaging studies were carried out on a dedicated RT table using fiducial body markers by experienced radiation therapy technologists trained specially for this purpose. The SPECT/CT fusion was always possible without major problems.

Gutman et al., recommended ^18^ F-FCH PET/CT in patients with a high risk of regional or metastatic disease [19]. Beheshti et al. revealed that the sensitivity and specificity of ^18^ F-FCH PET/CT in the detection of malignant LNs were 45 and 96%, respectively [20]. In our study we observed one suspicious regional LN metastasis in two patients, respectively. These two LNs with an abnormal ^18^ F-FCH uptake were not detected as SNs via SPECT, perhaps showing a limitation of SPECT. One hypothesis is that these small LNs could be heavily infiltrated by prostate cancer hampering the lymph path. A pathological evaluation of such lymph nodes might have confirmed this hypothesis. Although, ^18^ F-FCH PET/CT cannot yet be recommended as a standard diagnostic tool as part of the RT treatment planning in high-risk prostate cancer patients, the detection of these suspicious LNs in the pelvic region allowed to identify a target to be boosted with a high dose after the irradiation of the standard pelvic CTV. Further studies are required including extended pelvic lymph node dissection to evaluate the impact of combined ^18^ F-FCH PET/CT and SPECT/CT. Such studies should clarify if such multimodal imaging strategy leading to improvement in the detection of a geographic misses.

## Conclusion

Multimodality imaging combining SPECT/CT prostate lymphoscintigraphy and ^18^ F-FCH PET/CT identified SNs outside standard pelvic CTVs or highly suspicious pelvic LN metastases in a majority of high risk prostate cancer patients. Treatment planning was modified in 40% of patients due to the observation of pararectal SNs in 6 patients (30%) and a suspicion of LN metastases in PET/CT in 2 patients (10%) demonstrating the potential impact of this approach in RT treatment planning.

## Competing interests

The authors declare that they have no competing interests.

## Authors’ contributions

HV carried out the drafting of the manuscript and partially the analysis of the data. SN and MV carried out the phantom studies. RM carried out the design of the study. CS and FB carried out the evaluation of the SPECT/CTs and ^18^ F-FCH PET/CTs. AC participated in the methodological design and performed the ^99m^Tc-nanocolloid injection. TZ participated in the analysis of the data. GD carried out the RT planning evaluation. OR participated in the methodological design. All authors read and approved the final manuscript.
